# Evaluation of a Virus Neutralisation Test for Detection of Rift Valley Fever Antibodies in Suid Sera

**DOI:** 10.3390/tropicalmed4010052

**Published:** 2019-03-25

**Authors:** Baratang A. Lubisi, Phumudzo N. Ndouvhada, Donald Neiffer, Mary-Louise Penrith, Donald-Ray Sibanda, Armanda D.S. Bastos

**Affiliations:** 1 Agricultural Research Council – Onderstepoort Veterinary Institute, Onderstepoort 0110, South Africa; NdouvhadaP@arc.agric.za; 2 Mammal Research Institute, Department of Zoology & Entomology, University of Pretoria, Private Bag 20, Hatfield 0028, South Africa; Amanda.Bastos@up.ac.za; 3 Department of Agriculture and Animal Health, College of Agriculture and Environmental Sciences, University of South Africa, Private Bag X6, Florida 1710, South Africa; Donvet@gmail.com; 4 Wildlife Health Sciences, National Zoological Park, Smithsonian Conservation Biology Institute, P.O. Box 37012, Washington, DC 20013-7012, USA; NeifferD@si.edu; 5 Department of Veterinary Tropical Diseases, Faculty of Veterinary Science, University of Pretoria, Private Bag X04, Onderstepoort 0110, South Africa; marylouise@vodamail.co.za; 6 Centre for Veterinary Wildlife Studies, Department of Para-clinical Sciences, Faculty of Veterinary Science, University of Pretoria, Private Bag X04, Onderstepoort 0110, South Africa

**Keywords:** Rift Valley fever, Rift Valley fever virus, inter-epidemic period, domestic pig, ELISA and VNT

## Abstract

Rift Valley fever (RVF) is a vector-borne viral disease of ruminants mainly, and man, characterized by abortions and neonatal deaths in animals and flu-like to more severe symptoms that can result in death in humans. The disease is endemic in Africa, Saudi Arabia and Yemen, and outbreaks occur following proliferation of RVF virus (RVFV) infected mosquito vectors. Vertebrate animal maintenance hosts of RVFV, which serve as a source of virus during inter-epidemic periods remain unknown, with wild and domestic suids being largely overlooked. To address this, we evaluated the virus neutralization test (VNT) for RVF antibody detection in suid sera, as a first step in assessing the role of suids in the epidemiology of RVF in Africa. Testing of experimental and field sera from domestic pigs and warthogs with a commercial RVF competitive antibody ELISA, served as a reference standard against which the VNT results were compared. Results indicate that VNT can detect anti-RVFV antibodies within three days post-infection, has an analytical specificity of 100% and diagnostic sensitivity and specificity of 80% and 97%, respectively. Although labour-intensive and time-consuming, the VNT proved suitable for screening suid sera and plasma for presence of RVFV antibodies in viraemic and recovered animals.

## 1. Introduction

Rift Valley fever (RVF) is a vector-borne disease of ruminants, camels and man, characterized by widespread abortions, teratogenicity, and neonatal deaths in animals, and flu-like symptoms which can progress to severe disease and even death in humans [1,2]. The causal agent is RVF virus (RVFV), genus *Phlebovirus*, order Bunyavirales, and family Phenuiviridae [3]. 

The disease is endemic in Africa, Saudi Arabia and Yemen, and mosquitoes, primarily those of the *Aedes* and *Culex* genera, act as vectors and transmit RVFV from one host to the other. Unborn animal foetuses can contract infection transplacentally [4] and vertical transmission in humans has also been reported [5,6]. Outbreaks occur after periods of high rainfall or in environments supporting the proliferation of RVFV-infected mosquito vectors [7]. Up until recently, it was believed that transovarial transmission of RVFV by infected *Aedes* mosquitos allowed them to act as inter-epidemic period (IEP) reservoir hosts but current research indicates that whilst vertical transmission in mosquitoes is likely, there is insufficient evidence to support the hypothesis [8,9]. Serological evidence of low-level circulation of RVFV among wild and domestic animals during the IEP exists, but definitive mammalian reservoir hosts remain unidentified [10]. 

Due to its zoonotic nature, a “One health approach” involving a wide range of institutions and authorities with different expertise is usually adopted in response to outbreaks and when instituting preventative measures. Diagnosis of RVF employs various serological and agent identification methods such as antibody detecting ELISAs, as well as virus isolation (VI), virus neutralisation test (VNT), indirect immunofluorescent assays (IIFA), immunohistochemistry (IHC), and reverse transcriptase polymerase chain reaction (RT-PCR) [11]. While the search for IEP vertebrate maintenance hosts of RVFV for purposes of improved disease surveillance and control continues, it is imperative that the diagnostic tools used to analyse samples from species other than domestic ruminants are confirmed fit for the intended purposes.

We report here on the evaluation of a VNT capable of detecting RVFV antibodies in domestic and wild pig sera and plasma, implemented as part of a broader study investigating the potential role of suids in RVF epidemiology in Africa.

## 2. Materials and Methods

### 2.1. Viruses

Two genetically distinct RVFV strains from the 2009 (M259/09) and 2010 (M21/10) outbreaks in Northern Cape and Free State Provinces of South Africa respectively, were used to inoculate pigs and control lambs in viral infectivity studies conducted at the Agricultural Research Council - Onderstepoort Veterinary Institute (ARC–OVI)’s BSL 3 facility. The 2009 strain was isolated from bovine blood, passaged 5 and 15 times in BHK and Vero cells respectively, and used at a titre of 5 × 10^6^ PFU/100 µL, while the 2010 virus was obtained from an ovine organ pool, passaged 3 and 14 times in BHK and Vero cells respectively, and utilised at a titre of 0.5 × 10^4^ PFU/100 µL.

### 2.2. Animal Inoculations

All animal experiments were carried out using protocols approved by the ARC-OVI’s Animal Ethics Committee (AEC) under application number AEC 10.16 and endorsed by the University of Pretoria’s AEC (Ref. EC057-17). 

Pregnant and lactating sows, 1 to 2-day old suckling piglets and weaner piglets were procured from the ARC – Animal Production Institute (ARC-API) and housed at the ARC - OVI - BSL 3 experimental animal facility. The animals were divided into 2 groups. Group 1 consisted of 1 lactating and 5 pregnant sows, 10 suckling piglets and 9 weaners (*n* = 25), and group 2 constituted 4 pregnant and 2 lactating sows, 20 suckling piglets and 9 weaners (*n* = 35). Viruses M259/09.5BHK.15Vero and M21/10.3BHK.14Vero at their respective titres in 2 ml volumes were used to inoculate pregnant sows, suckling piglets and weaners of each group via the jugular vein.

Two suckling lambs of approximately 4 days were challenged in each group and used as controls. The animals were bled from the jugular vein using vacutainer tubes and standard methods from 0–7 days post infection (dpi), and at 14, 21, 28, 30 and 61 dpi. Newly born piglets were also bled. All sera were stored at 4 degrees Celsius until further use. 

### 2.3. Sera

All sera used in the study were screened for RVFV antibodies with a commercial competitive ELISA (ID Screen^®^) which has multispecies application. Since RVF is not a pig disease and sources of known positive sera were unavailable, porcine sera produced in the above experiments and field porcine and warthog samples were used for evaluation of analytical sensitivity (ASe), analytical specificity (ASp), diagnosic sensitivity (DSe) and diagnostic specificity (DSp). Cross reactivity studies could not be conducted due to lack of other *Bunyaviruses* and corresponding antisera (Table 1). 

### 2.4. Additional Performance Measures

Apart from analytical and diagnostic performance, the VNT was assessed for: (i) influence of potentially inhibitory factors in serum arising from haemolysis and putrefaction (*n* = 2); (ii) repeatability, where experimental sera (*n* = 2) were tested in replicates of 4 in 5 plates for 7 consecutive days; (iii) robustness, through first incubation of replicate test plates for different time periods (60, 90 and 120 min), using different test virus titres (100–300 and 1000 TCID_50_/mL) in replicate plates, and further incubating the plates 1–3 days following recording of results; and (iv) reproducibility where inter-analyst comparison (*n* = 84) and inter-method comparison with an in-house IgG indirect ELISA (*n* = 119) were performed [12]. 

### 2.5. Serological Tests

#### 2.5.1. ELISA 

The ID Screen^®^ Rift Valley Fever Competition Multi-species ELISA (ID-VET, Montpellier, France) intended for detection of both IgM and IgG anti-Rift Valley Fever (RVF) antibodies in ruminant serum or plasma was used as the standard of comparison for the VNT. The test is reported to have a diagnostic sensitivity and specificity of 98% and 100% respectively [13]. Testing was performed according to the manufacturer’s instructions and results were read at a wavelength of 450 nm. 

#### 2.5.2. Virus Neutralisation Test

The VNT was conducted as described previously with a few modifications [14]. Test sera were heat inactivated at 56 °C for 30 min and allowed to cool. Initial 1/5 dilutions in DMEM (Lonza, Switzerland) containing NEAA, Penicillin, Streptomycin and Ampotericin B, were loaded on 96 well plates in duplicate and subsequent two-fold dilutions made down the plates. Virus M259/09 5BHK.15Vero described previously was added at titres of 100–300TCID_50_ to each well containing serum, and the plates were incubated at 37 °C for 1 h in a humid chamber with 5% CO_2_. Vero cells (ATCC, USA) at a concentration of 3 to 4 × 10^5^ cells per ml were added and the plates were incubated at 37 °C for 3–5 days. Cell, virus titration, sample, and control sera plates were included with each test run, with reference ovine RVFV (strain 35/74) antiserum and serum with undetectable RVFV antibodies by in-house indirect IgG and IgM capture ELISAs used as positive and negative control sera, respectively [12]. 

The plates were monitored daily under an inverted microscope and when the control virus showed cytopathic effect (CPE) of 90%–100%, presence of CPE and intact cell monolayers was recorded and scored. For confirmation of results, plates were fixed with 10% formalin containing 0.05% crystal violet, and re-visualised using the microscope. Serum antibody titres were taken as the reciprocal of the dilution at which presence of either no (0%) or minute CPE (~10%) was observed.

### 2.6. Statistics

Association, differences and agreement between the competitive ELISA and VNT were determined using Chi-square and Fisher’s exact test, McNemar Chi-square, and Cohen’s kappa coefficient (κ) respectively, using GraphPad software version 8.0.1.244 [15,16]. Receiver operating characteristic (ROC) curve analysis was utilised to select the best VNT positive cut-off value and the graph was drawn using Microsoft Excel 2016 [15,17]. MedCalc Software version 18.5 was used to evaluate test performance [18]. Confidence intervals for all calculations were set at 95% 

The VNT would be deemed fit for the intended purpose if results were repeatable, reproducible and statistically and significantly in agreement with those of the competitive ELISA.

## 3. Results

### 3.1. Operating Range and Thresholds

**Figure 1:**
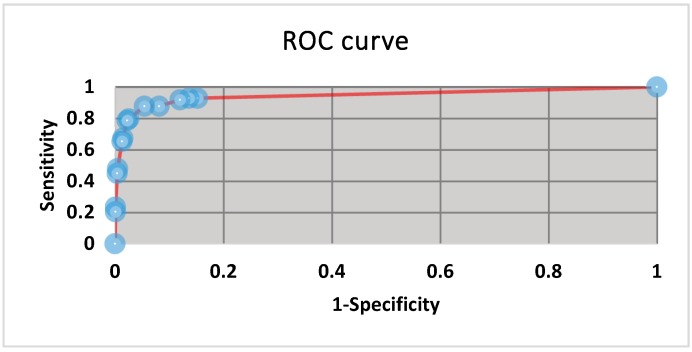
Receiver operating characteristic curve generated from 14 cut-off values, indicating the best positive cut off values to be between 1/60 and 1/80 dilutions. The area under the curve is 0.94.

The minimum and maximum serum dilutions at which anti-RVFV antibodies could be detected by the VNT were 1/5 to 1/1280 according to the titre of the positive control serum used. The serum dilution at which inherent toxins and haemolysis products had cleared and cell monolayer integrity and visibility was intact in more than 90% of the samples tested was at dilutions above 1/40. The best positive cut-off was set at 1/60 following ROC curve analysis and the area under the curve (AUC) was 0.94 (CI: 80%–100%) (Figure 1). Suspect titre was set at 1/40, which was the dilution at which known positive sera would show protection when the plates were over incubated or high test-virus titres were used. 

### 3.2. Analytical Sensitivity and Specificity

Anti-RVFV antibodies could be detected in swine sera as early as 3 dpi by both ELISA and VNT. Positive results were obtained at different dilutions according to the antibody titres of the sera tested, and 1/640 was the lowest dilution at which positives were recorded. All pre-inoculation samples tested negative with both ELISA and VNT, thus analytical specificity (ASp) was 100% (CI: 92%–100%). 

### 3.3. Diagnostic Sensitivity and Specificity

For purposes of calculating diagnostic sensitivity (DSe) and diagnostic specificity (DSp), the IDVET competitive ELISA suspect results were regarded as positive and those of the VNT were taken as negative. Diagnostic sensitivity and specificity were 80% (CI: 70% to 87%) and 97% (CI: 96% to 98%), respectively.

### 3.4. Additional Performance Measures

Putrefied sera and those with products of haemolysis were cytotoxic and resulted in cell lysis and unclear visibility of the cell monolayers. Intra-plate and inter-plate variability measured as percent coefficient of variation (%CV) ranged from 0%–30% and 5.7%–30% respectively, with inter-plate variability increasing above 30% only when incubation times were extended past 3–5 days and test virus titres differed by one log between plates. The %CV of the in-house indirect ELISA and VNT, and VNT inter-analyst results were 1.8% and 2.4%, and 2.8% and 3%, respectively. 

### 3.5. Statistics and Fitness for Purpose

Analysis of association between the results of the ELISA and VNT using Chi-square and Fisher’s exact test yielded a two-tailed *p*-value < 0.0001. A total of 52 insignificant (*p* = 0.12) discordant results were discerned by the McNemar test, and the odds ratio was 1.600 (CI: 0.89 to 2.95). Number of agreements between the test classifications were 1300 (96.15%) and Kappa was 0.78 (CI: 0.66–0.8).

## 4. Discussion

In the absence of validated serological tests for use in non-target species, highly specific neutralisation tests are employed instead. The neutralisation method currently listed in the RVF chapter of the Manual of Diagnostic Tests and Vaccines for Terrestrial Animals of the Office International des Epizooties (OIE) is the plaque reduction neutralisation test (PRNT) [11].

The PRNT method allows the infective virus to cause CPE slowly and is thus ideal for quantifying the starting virus [19]. However, the method is more time-consuming, expensive and labour-intensive than the VNT [20]. Some of the drawbacks include: (i) fewer samples per plate can be tested compared to the VNT and larger sample volumes, which may not be available, are required; (ii) cell-seeding and addition of dyes prior to commencement of the test and reading of the plates respectively, are needed, which adds two additional days to the test procedure; (iii) gel-based medium overlays such as agarose require heating and can cause damage to cell monolayers and negatively affect heat-labile viruses if not kept at optimum temperatures; (iv) dyes such as neutral red are photosensitive, have a short shelf life and can crystallize and interfere with the test; and (v) because many virus strains have pinpoint-sized plaque phenotypes, result interpretation is difficult and must be performed by highly trained personnel [20].

While PRNT is sensitive and highly specific, it is not ideal for resource-poor laboratories whose primary intentions are to screen large numbers of field sera for RVFV antibodies following vaccination campaigns, when disease incursion is suspected or for surveillance during IEP. Since various forms of neutralisation assays are regarded as the “gold standard” when evaluating or validating serological assays, another method with high DSe and DSp was required when evaluating the suitability of VNT for RVFV antibody detection in suid samples. The ID Screen^®^ RVF antibody competitive ELISA (ID-VET, Montpellier, France) was selected because it has a multi-species application, is reasonably validated, widely used and commercially available [12,13,21,22].

As previously hypothesized, domestic pigs can be successfully infected with high doses of RVFV [23], sero-convert, shed virus in their secretions and vertically transmit the virus to their offspring in-utero, as attested by demonstration of RVFV antibody, RNA, antigen, virions, and viable virus in tissue samples of sows, piglets and offspring of experimentally infected sows [24]. Due to unavailability of positive field samples, suid sera from the experimental infections had to be utilised instead. A limitation to the study was that experimental sera collected 0-7dpi were only analysed for antibody presence omitting analysis by PCR to confirm successful infection, due to resource constraints. In addition, the number of experimental animals was out of necessity kept at a minimum with samples collected from individual pigs on different days being used to assess diagnostic performance, thus impacting sample independence. We were nonetheless able to recover an association between the outcomes of the ELISA and VNT. Agreement between the sample status classification (96.15%) was statistically significant (*p* < 0.0001) and strong (K = 0.78), and the observed differences (3.85%) were regarded as insignificant. Repeatability and reproducibility of the VNT was good at %CV between 0%–30% and an AUC above 0.9 confirmed the robustness of the test for dichotomous discrimination of samples.

**Figure 2:**
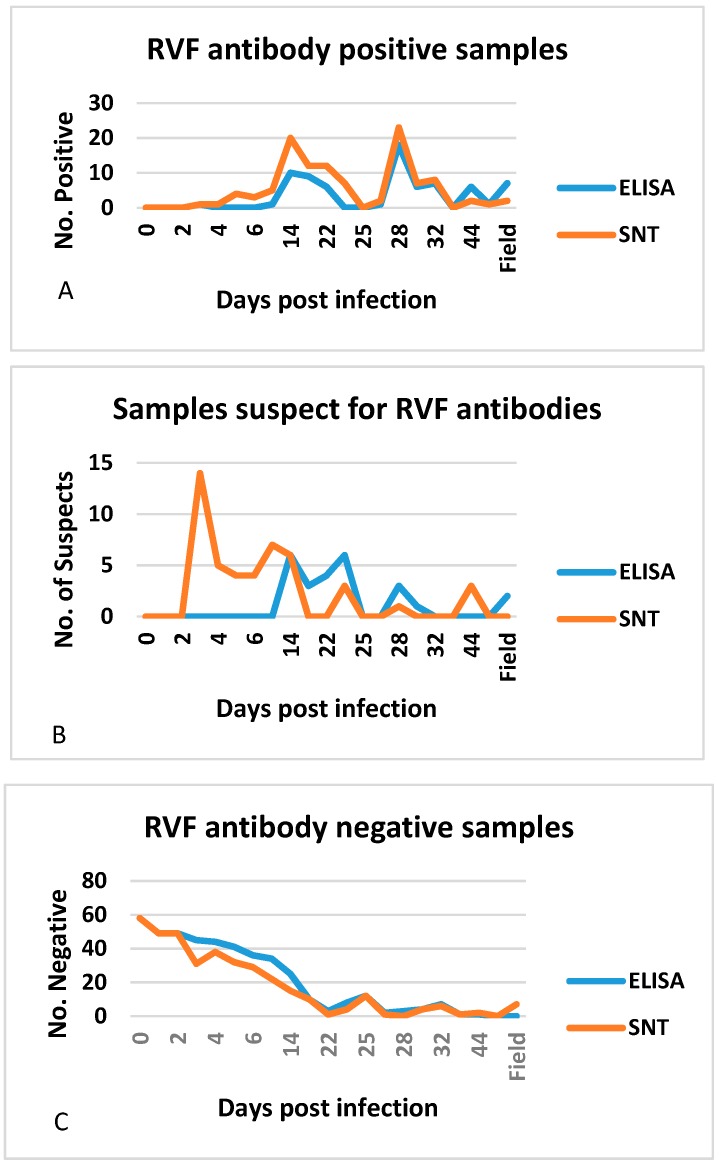
Number of RVF antibody positive (**A**), suspect (**B**) and negative (**C**) experimental and field sera detected by competitive ELISA and VNT. The experimental samples were constituted by sera from sows, weaners and suckling pigs, and piglets born from virus inoculated pregnant sows between dpi 0 and 61. Only discordant field sera are represented in the graphs.

Assessment of the two tests without regarding either as the standard of comparison and taking into consideration their respective cut-off values, showed that the VNT classified more samples as positive and suspect than the ELISA (Figure 2), and that the level of agreement of the results (92%) was high (Table 2). The competitive ELISA and VNT are purported to be capable of detecting both RVFV IgM and IgG antibodies, with the IgM usually appearing within the first week of infection [25]. For the experimentally infected animals, the VNT yielded suspect (*n* = 34) and positive (*n* = 14) results from dpi 3 to 7, while the ELISA only yielded two positive (one each on dpi 3 and 7), and no suspect results. The majority of positive results were detected from dpi 14 for both methods. Only 1/9 ELISA-positive field sera were designated as such by the VNT, probably due to low titres.

Suspect results would normally be re-tested using other methods or the donor animals re-bled, thus positives are unlikely to be missed. Of concern is the negative classification of several sera from experimentally infected animals between dpi 3 and 14 by the competitive ELISA, as utilisation of a method with low sensitivity for IgM antibodies, would result in positive and viraemic animals being missed and in the potential for disease to spread. 

Domestic pigs are closely related to humans in terms of anatomy, physiology and genetics, and can serve as excellent animal models to study human infections [26]. In the event of using pigs to study RVF to generate new knowledge for improved animal and human health, it is imperative to evaluate the suitability of diagnostic tools for use in this species. The results of the VNT proved statistically agreeable with those of the competitive ELISA used for comparative purposes in this study. Both methods proved suitable for screening suid sera and plasma for RVFV antibodies in experimental and field studies. However, as the VNT detected more positive samples than the ELISA in experimentally infected pigs, especially during early infection, it is, in the absence of better alternatives, the preferred method for detecting RVF antibodies in suids.

## Figures and Tables

**Table 1:** Experimental and field samples categorised as positive and negative by the competition ELISA and used in the evaluation of the virus neutralisation test (VNT). The samples used for determination of analytical performance were included in sessing assay diagnostic capability.

Purpose	Animals	Source	Antibody Status	Days Post Infection	Number Animals: Number of Samples
**Analytical specificity**	Sows	Experimental	Negative	Pre-infection	11:11
	Weaners	Experimental	Negative	Pre-infection	27:27
	Suckling piglets	Experimental	Negative	Pre-infection	20:20
**Total**					58:58
**Analytical sensitivity**	Weaners	Experimental: Infected with M21/10 RVFV	Positive	3, 7, 14, 21 and 28	5:5
	Newborn piglets	Experimental: Dams infected with M21/10 and M259/09 RVFV	Positive	22, 28, 32, and 44	4:4
	Sow	Experimental: Infected with M259/09 RVFV	Positive	61	1:1
**Total**					10:10
**Diagnostic sensitivity**	Suckling piglets	Experimental: Infected with M21/10 and 259/09 RVFV	Positive	14	2:2
	Newborn piglets	Experimental: Dams infected with M21/10 and M259/09 RVFV	Positive	22, 23, 28, 32, 44	41:41
	Weaners	Experimental: Infected with M21/10 and 259/09 RVFV	Positive	3, 7, 14, 21, 27, 28, 30	16:30
	Sows	Experimental: Infected with M21/10 and 259/09 RVFV	Positive	14, 21, 27, 28, 61	7:16
	Porcine: various ages	Field	Positive	N/A	6:6
	Warthogs: Mixed ages	Field	Positive	N/A	3:3
**Total**					75:98
**Diagnostic specificity**	Suckling piglets	Experimental: Infected with M21/10 and 259/09 RVFV	Negative	0– 7, 14, 21	20:132
	Newborn piglets	Experimental: Dams infected with M21/10 and M259/09 RVFV	Negative	22, 23, 25, 32, 44	29:29
	Weaners	Experimental: Infected with M21/10 and 259/09 RVFV	Negative	0–7, 14, 21, 28, 30	27:167
	Sows	Experimental: Infected with M21/10 and 259/09 RVFV	Negative	0–7; 14, 21, 22, 27, 34	12:104
	Porcine: various ages	Field	Negative	N/A	725:725
	Warthogs: Mixed ages	Field	Negative	N/A	97:97
**Total**					910:1254
**Grand Total**					985:1352

N/A: not applicable.

**Table 2:** Comparison of the level of agreement between the competition ELISA and VNT in Rift Valley fever (RVF) antibody status classification of experimental and field sera.

-	-	ELISA			-
		Positive	Suspect	Negative	
**VNT**	Positive	61 (4.5%)	17 (1.3%)	32 (2%)	110 (8%)
	Suspect	6 (0.5%)	2 (0.15%)	39(3%)	47 (3.5%)
	Negative	6 (0.5%)	6 (0.5%)	1183 (88%)	1195 (88.5%)
		73 (5.5%)	25 (2%)	1254 (93%)	1352
